# Neurological update: non-motor symptoms in atypical parkinsonian syndromes

**DOI:** 10.1007/s00415-023-11807-x

**Published:** 2023-06-15

**Authors:** Piriyankan Ananthavarathan, B. Patel, S. Peeros, R. Obrocki, N. Malek

**Affiliations:** 1 Department of Neurology, National Hospital for Neurology and Neurosurgery, Queen Square, London, UK; 2 Department of Neuroinflammation, Institute of Neurology, University College London, 1st Floor, Russell Square House, 10-12 Russell Square, London, WC1B 5EH UK; 3 Department of Neurology, Queen’s Hospital, Romford, Essex UK

**Keywords:** Parkinson’s disease, Atypical parkinsonism, Non-motor symptoms, Progressive supranuclear palsy, Multiple system atrophy, Corticobasal degeneration, Dementia with Lewy bodies

## Abstract

Among people with Parkinson’s disease (PD), non-motor symptoms (NMS) are a well-recognised cause of significant morbidity and poor quality of life. Yet, it is only more recently that NMS have been recognised to affect the lives of patients with atypical parkinsonian syndromes in a similar fashion. The aim of this article is to highlight and compare the relative prevalence of NMS among patients with atypical parkinsonian syndromes in the published literature, which largely remain underreported and unaddressed in routine clinical practice. All NMS that are recognised to occur in PD are also found to commonly occur in atypical parkinsonian syndromes. In particular, excessive daytime sleepiness is more prevalent among atypical parkinsonian syndromes (94.3%) compared to PD (33.9%) or normal controls (10.5%) (*p* < 0.001). Urinary dysfunction (not limited to urinary incontinence) is not only found to occur in MSA (79.7%) and PD (79.9%), but has also been reported in nearly half of the patients with PSP (49.3%), DLB (42%) and CBD (53.8%) (*p* < 0.001). Apathy is significantly more common among the atypical parkinsonian syndromes [PSP (56%), MSA (48%), DLB (44%), CBD (43%)] compared to PD (35%) (*p* = 0.029). Early recognition and addressing of NMS among atypical parkinsonian syndromes may help improve the holistic patient care provided and may encompass a range of conservative and pharmacotherapeutic treatments to address these symptoms.

**Supplementary Information:**

The online version contains supplementary material available at 10.1007/s00415-023-11807-x.

## Introduction

Atypical parkinsonian syndromes, including multiple system atrophy (MSA), progressive supranuclear palsy (PSP), corticobasal degeneration (CBD) and dementia with Lewy bodies (DLB), have a wide, yet overlapping, phenotypic spectrum of motor and non-motor symptoms [1, 2], which have been recognised almost since the earliest descriptions of the disorders [1]. Non-motor symptoms (NMS) are notably present in both the prodromal phases of Parkinson’s disease (PD) [3] and pre-motor phases of atypical parkinsonism [4]. Even when not present initially, most patients with atypical parkinsonism invariably experience many NMS with disease progression, which have significant effects on quality of life [5], disability, morbidity and mortality [6, 7]. One clinicopathological case series [8] demonstrated NMS (including behavioural and psychiatric symptoms) preceded motor symptoms in nearly half of the autopsy-confirmed cases of CBD. Owing to the involvement of several neural circuits beyond the basal ganglia, there is an ongoing debate as to whether NMS are actually more prominent among atypical parkinsonian disorders compared to PD [2, 9]. Yet, research into the true prevalence of NMS among atypical parkinsonian syndromes has been limited compared to PD [1].

Over the last decade though, several research groups have attempted to systematically evaluate the presence and severity of NMS in atypical parkinsonian syndromes, most of which overlap with that experienced by patients with PD. Along with strongly correlating with motor complications in PD [10], the presence of NMS, mood, and gait abnormalities have been shown to significantly contribute to the poor quality of life experienced by patients with PD [11]. This finding is also mirrored amongst patients with atypical parkinsonian syndromes, where cognitive impairments and depressive symptoms have been shown to be strongly linked to lower quality-of-life scores [12, 13].

Despite similarities in the range of NMS observed among patients with PD and atypical parkinsonian syndromes, there are clear differences in the relative prevalence of individual NMS [2] in the different atypical parkinsonian syndromes. Yet, most NMS remain largely underreported by patients with atypical parkinsonian syndromes and are therefore unaddressed during assessments in routine clinical practice. The aim of this article is to highlight and discuss the prevalence of NMS in atypical parkinsonian syndromes as published in the scientific literature over the past 30 years (Tables 1, 2, 3, 4).

**Table 1:** Prevalence of non-motor symptoms in progressive supranuclear palsy

Author (year)	Constipation	Depression	Hallucinations	Delusions	Apathy	Anxiety	RBD	OH	EDS	Anosmia	Urinary dysfunction	Drooling	Sexual dysfunction
Schrag (2003) [12] (*n* = 27)	Not Reported	14	Not reported	Not reported	6	8	Not reported	Not reported	Not reported	Not reported	Not reported	Not reported	Not reported
O’Sullivan (2008) [32] (*n* = 110)	Not reported	Not reported	Not reported	Not reported	Not reported	Not reported	Not reported	Not reported	Not reported	Not reported	27	Not reported	Not reported
Reimann (2009) [72] (*n* = 32)	14	Not reported	Not reported	Not reported	Not reported	Not reported	Not reported	5	Not reported	Not reported	25	6	Not reported
Colosimo (2010) [2] (*n* = 30)	24 (grouped as GI symptoms)	22 (grouped as psychiatric disorders)	22 (grouped as psychiatric disorders)	22 (grouped as psychiatric disorders)	20	22 (grouped as psychiatric disorders)	23 (grouped as sleep dysfunction)	4 (grouped with falls)	23 (grouped as sleep disturbance)	Not reported	12	24 (grouped as GI symptoms)	Not reported
Srulijes (2011) [17] (*n* = 23)	Not reported	Not reported	Not reported	Not reported	Not reported	Not reported	Not reported	Not reported	Not reported	not reported	Not reported	Not reported	Not reported
Higginson (2012) [64] (*n* = 12)	9	Not reported	2	Not reported	Not reported	Not reported	Not reported	Not reported	11	Not reported	7	Not reported	Not reported
Ou (2016) [18] (*n* = 27)	23 (grouped with hypersalivation difficulty swallowing)	27 (grouped with apathy)	4 (grouped with delusions)	4 (grouped with delusions)	27 (grouped with depression)	Not reported	Not reported	5 (grouped with falls)	27	Not reported	20	23 (grouped with dysphagia and constipation	25
Pellicano (2017) [16] (*n* = 25)	Not reported	16	Not reported	Not reported	6	1	Not reported	Not reported	Not reported	Not reported	Not reported	Not reported	Not reported
Radicati (2017) [19] (*n* = 50)	29	24	18 (grouped with delusions)	18 (grouped with hallucinations)	44 (grouped with depression)	Not reported	Not reported	25 (grouped with falls)	46	Not reported	46	43 (Grouped with DYSPHAGIA and constipation)	9
Lee (2018) [71] (*n* = 19)	Not reported	Not reported	Not reported	Not reported	Not reported	Not reported	Not reported	Not reported	Not reported	Not reported	1	Not reported	Not reported
Santangelo (2018) [15] (*n* = 42)	Not reported	9	Not reported	Not reported	16	Not reported	Not reported	Not reported	Not reported	Not reported	Not reported	Not reported	Not reported

**Table 2:** Prevalence of non-motor symptoms in Multiple System Atrophy

Author (*n*)	Constipation	Depression	Hallucinations	Delusions	Apathy	Anxiety	RBD	Orthostatic Hypotension	EDS	Anosmia	Urinary Dysfunction	Drooling	Sexual Dysfunction
Lee (2018) [5] (*n* = 14)	Not reported	Not reported	Not reported	Not reported	Not reported	Not reported	Not reported	Not reported	Not reported	Not reported	6	Not reported	Not reported
Reimann (2010) [72] (*n* = 38)	14	Not reported	Not reported	Not reported	Not reported	Not reported	Not reported	5	Not reported	Not reported	25	6	Not reported
Higginson (2012) [64] (*n* = 17)	13	Not reported	5	Not reported	Not reported	Not reported	Not reported	Not reported	14	Not reported	12	Not reported	Not reported
Santangelo (2018) [15] (*n* = 42)	Not Reported	6 Depression alone 15 Depression and apathy	Not reported	Not reported	6	Not reported	Not reported	Not reported	Not reported	Not reported	Not reported	Not reported	Not reported
Ha (2011) [73] (*n* = 26)	Not reported	Not reported	Not reported	Not reported	Not reported	Not reported	Not reported	21	Not reported	Not reported	Not reported	Not reported	Not reported
Barcelos (2018) [33] (*n* = 14)	Not reported	Not reported	Not reported	Not reported	Not reported	Not reported	Not reported	8	Not reported	Not reported	Not reported	Not reported	Not reported
Colosimo (2009) [2] (*n* = 34)	28 (grouped as GI symptoms)	27 (grouped as psychiatric disorders)	27 (grouped as psychiatric disorders)	27 (grouped as psychiatric disorders)	22	27 (grouped as psychiatric disorders)	23 (grouped as sleep disturbance)	18	23 (grouped as sleep disturbance)	Not reported	30	28 (grouped as GI symptoms)	Not reported
Du (2018) [13] (*n* = 143)	105	Not reported	Not reported	Not reported	Not reported	Not reported	Not reported	43	Not reported	Not reported	114 Nocturia 112 Urinary urgency 100 Urinary incontinence	101	Not reported
Zhang (2016) [65] (*n* = 172)	94	127	6	1	115	78	Not reported	120 (grouped as lightheaded-ness)	91	40 (grouped with taste)	157	51	125

**Table 3:** Prevalence of non-motor symptoms in Corticobasal degeneration

Author (year)	Constipation	Depression	Hallucinations	Delusions	Apathy	Anxiety	RBD	Orthostatic hypotension	EDS	Anosmia	Urinary dysfunction	Drooling	Sexual Dysfunction
Colosimo (2010) [2] (*n* = 11)	7	6 (grouped as psychiatric disorders)	6 (grouped as psychiatric disorders)	6 (grouped as psychiatric disorders)	7	6 (grouped as psychiatric disorders)	Not reported	0	4 (grouped as sleep disturbance)	Not documented	6	Not reported	Not reported
Ikeda (2014) [8] (*n* = 9)	Not reported	3	2 (1 visual, 1 auditory)	1	2	Not reported	Not reported	Not reported	Not reported	Not reported	Not reported	Not reported	Not reported

**Table 4:** Prevalence of non-motor symptoms in dementia with Lewy bodies

Author (year)	Constipation	Depression	Hallucinations	Delusions	Apathy	Anxiety	RBD	Orthostatic hypotension	EDS	Anosmia	Urinary dysfunction	Drooling	Sexual Dysfunction
Chiba (2012) [66] (*n* = 34)	16	8	Not reported	Not reported	9	9	21 Sleep rhythm change 21 Crying/shouting 12 Limb movements 9 Nightmares	8	Not reported	14	9	7	Not reported
Colosimo (2010) [2] (*n* = 14)	12 (grouped as GI symptoms)	13 (grouped as psychiatric disorders)	13 (grouped as psychiatric disorders)	13 (grouped as psychiatric disorders)	12	Not reported	11 (grouped as sleep disturbance)	3	Not reported	Not reported	11	Not reported	Not reported

## Overall burden of NMS

NMS, including cognitive, behavioural, sleep and autonomic symptoms are seen in all atypical parkinsonian syndromes, where they not only affect a patient’s wellbeing but also the quality of life of their caregivers [14]. Several research groups have attempted to systematically assess the prevalence and burden of NMS in various atypical parkinsonian syndromes [2, 5, 15–19], while some have attempted to also compare the relative burden of NMS in different atypical parkinsonian syndromes and PD. One group of researchers [5], used the total non-motor symptom scale (NMSS) scores to categorise patients with MSA (*n* = 43), PSP (*n* = 14) and PD (*n* = 117) into 5 stages as a proposed mechanism to compare their NMS burdens [20] [for scoring, stage 0: 0; stage 1: 1–7; stage 2: 8–24; stage 3: 25–44; stage 4: 44–80; stage 5: > 81], and found that while NMSS staging did not vary significantly between cases, a greater proportion of patients with PSP (64%) had a higher NMSS stage (4 or 5), compared to those with MSA-P (48%), MSA-C (58%) and PD (47%) [5]. Another comparative study [19] evaluating NMS burden using NMSS in 50 PSP and 100 PD patients, similarly also found patients with PSP had significantly worse total NMSS scores compared to patients with PD (*p* < 0.0001). A more recent study from India [21] compared NMS in patients with either atypical parkinsonism or PD and also showed that the burden of NMS was significantly worse among those with atypical parkinsonian syndromes. In this study, involving 188 participants (101 PD, 42 PSP, 45 MSA), the mean NMSS score was 37.1 ± 33.1 among patients with PD, 70.7 ± 50.7 in those with PSP, and 80.0 ± 43.7 among those with MSA (*p* < 0.001) [21].

## Cognition

Cognition is affected in all atypical parkinsonian syndromes to varying degrees, though most prominently among those with PSP, DLB and CBD.

### PSP

Early cognitive dysfunction is one of the key clinical features of PSP, distinguishing it from other atypical parkinsonian syndromes, wherein patients may present symptomatically up to three years before diagnosis. [22]

One British study [23] explored whether scores from the Addenbrooke’s Cognitive Examination-revised (ACE-R) could be used to clinically differentiate between patients that presented with either PD (*n* = 86) or other atypical Parkinsonian syndromes (*n* = 30 PSP, *n* = 19 CBD). Total ACE-R scores and subscores for verbal fluency and visuospatial domains distinguished patients with PSP from those with PD. In particular, verbal fluency most strikingly distinguished those with PSP, supporting the hypothesis that executive cognitive dysfunction appears to be an early prominent feature among those with PSP.

In another series of papers [24, 25], researchers reported on the cognitive outcomes among 124 patients with frontotemporal lobar degeneration syndromes (35 PSP, 29 CBS, 33 primary progressive aphasia (PPA) and 27 behavioural variant-FTD (bvFTD)) as part of an epidemiological study encompassing assessments with ACE-R, mini-mental state examination (MMSE), Frontotemporal Dementia Rating Scale (FRS), PSP Rating Scale (PSPRS) and Frontal Assessment Battery (FAB). Apathy, considered by some to be closely linked to executive dysfunction, was found to be a prominent clinical feature among patients with PSP and a strong predictor of survival among this cohort. Furthermore, when carer-rated changes in terms of everyday skills, self-care, motivation, and impulsive-apathetic or challenging behaviours were correlated against neuroimaging parameters, they appeared to demonstrate disruption in ventral frontotemporal tracts. [26]

Speech impairments are prominent among patients with PSP, particularly those with the PSP-cortical subtypes [27]. A smaller clinicopathological case series (*n* = 4) [28] initially reported the association of prominent features of speech apraxia and PNFA in pathologically-proven PSP cases. A year later, this was confirmed in a larger clinicopathological case series [29] from the same research group involving over 100 cases of frontotemporal lobar dementia, CBD and PSP, in which 10% of those with PSP were found to have been clinically diagnosed with apraxia of speech and PNFA. In another large, multicentre clinicopathological case series [30], 30% of patients with a tauopathy (of whom, 55% of patients had either a diagnosis of CBD or PSP) presented with clinical features of progressive aphasia. In yet another study [31], investigating speech and language impairments among patients diagnosed with either speech apraxia or PNFA, 11 patients were found to have either CBD or PSP. Among those where speech apraxia was predominant, the most likely diagnosis was PSP, while CBD was more likely among those where aphasia was the main speech abnormality.

One study conducted in China [18] observed deficits in memory and attention among 96% of patients with PSP, which was markedly higher than a European multicentre study [19] reporting a prevalence of 76%. Another comparative study [32] considered 110 patients with PSP and 83 with MSA of similar disease durations, and demonstrated cognitive impairments were significantly more likely to occur among those with PSP compared to MSA (*p* = 0.03). In another European multicentre study [2], the prevalence of cognitive problems was compared in patients with either vascular parkinsonism (VP) (*n* = 83), MSA (*n* = 34), PSP (*n* = 30), DLB (*n* = 14) and CBD (*n* = 11), where it was found that 63% of patients with PSP had attentional/memory problems using NMSS. Another comparative study [15] matched 20 patients with PSP by age, education and global cognitive status to 20 with PD, 20 with MSA and 20 with PSP, and found that those with PSP were more impaired on cognitive tests assessing executive function than any other group. Using semi-structured interviews and the Quality of Life Assessment Schedule (QOLAS) interview questionnaires, one British study [12] reported 38% of 27 patients with PSP reported concentration difficulties, while 33% reported memory impairments.

### MSA

In one European multicentre study [2] including 34 patients with MSA, 68% were found to have attentional/memory problems on NMSS. Meanwhile, another group [33] explored the prevalence of cognitive impairments in relation to postural hypotension among 10 MSA-P and 4 MSA-C patients and reported no significant differences in cognitive impairments between patients with or without postural hypotension.

### CBD

In relation to cognitive impairments in CBD, there appears to be a wide range of clinical presentations which patients may develop throughout their clinical course. Using the ACE-R, several researchers have shown patients with CBD demonstrate a broad range of cognitive impairments that affect multiple domains [34–38]. One Italian multicentre study [2] found that among 11 patients with a clinical syndrome of CBD, 70% reported attentional/memory problems.

A recent clinicopathological case series [39] considered the clinical course among 15 patients with a pathologically-confirmed diagnosis of CBD presenting with cognitive-predominant features (who had received a clinical diagnosis of either AD or FTD), and compared them to matched patients who were clinically diagnosed with CBS. At initial presentation, aphasia was found among 33% (5/15) with pathologically-confirmed CBD in this cases series, compared to 20% (3/20) in those clinical diagnosed with CBS (although the difference was not statistically significant) [39]. Meanwhile, apathy was far more common in those with pathologically-confirmed CBD (53%) compared to those who received a clinical diagnosis of CBS (16%) (*p* = 0.014) [39].

While executive dysfunction and memory problems are particularly common in CBD [34–37, 40], they may not always help distinguish CBD from other neurodegenerative conditions [41]. However, there appears to be a growing understanding of how impairments in language processing, visuospatial dysfunction and social cognition may be a possible distinguishing feature among patients with CBD [35, 40]. Speech and language dysfunction in particular appears to be a common presenting feature among patients with CBD [42, 43], where it has been estimated to occur in up to a third of patients [44–50], and encompass a wide range of clinical presentations including non-fluent aphasia [51], apraxia of speech [52] or agraphia [53]. In the large clinicopathological case series previously mentioned [29], 19% of patients with CBD were found to have clinical features of apraxia of speech and progressive non-fluent aphasia. In a Canadian case series [54] reporting on the evolution of several neurodegenerative conditions, a handful of cases that met the clinical diagnostic criteria for primary progressive aphasia were subsequently found to have CBD pathology at post-mortem.

Visual dysfunction appears to be another common problem among patients with CBD [37, 42, 45, 48, 51, 55, 56], and is included in the diagnostic criteria for the disease [51, 57, 58]. Although several studies [37, 45, 48] did not find any gross visual impairments on formal testing, one review [41] discussed how this might be due to confounding factors such as limb apraxia, motor deficits or co-existent executive dysfunction which could limit the ability to formally test visuospatial function.

Behaviour and personality changes have also been reported in the literature to occur commonly among patients with CBS [35, 40, 42, 59, 60], yet there appear to be few clinicopathological studies that comprehensively explore this aspect further. In one Mayo clinic study that was discussed [39], behavioural changes were reported to occur among 93% of a pathologically confirmed cohort of CBD who presented with cognitive impairment, compared to 19% in those with clinically diagnosed CBS (*p* < 0.001) [39].

### DLB

Dementia defined as a progressive cognitive decline of sufficient magnitude to interfere with either normal social or occupational function, or with usual daily activity, is an essential criterion for diagnosing DLB. Fluctuating cognition is one of the core criteria in making the diagnosis [61], yet may not be exhibited by all patients as evidenced by one study reporting the phenomenon to occur in less than half of patients (44.5%) [62].

## Behavioural and neuropsychiatric manifestations

Neuropsychiatric symptoms can be encountered in all forms of atypical parkinsonism but appear to be most prominent among patients with DLB.

### PSP

Mood [5] appears to have a significant correlation with the quality of life scores (based on PD-Q 39) in patients with PSP (*ρ* = 0.744, *p* = 0.002). Compared to healthy controls, all patients with PSP in one study from western China exhibited problems with mood/apathy, while 15% of patients with PSP also demonstrated hallucinatory or perceptual disturbances [18]. The frequency and severity of mood/apathy and attention/memory symptom domains on NMSS were significantly higher among patients with PSP compared to healthy controls (*p* < 0.05), while mood/apathy and attention/memory symptoms were also significantly higher in patients with PSP than those with PD (*p* < 0.05) [18]. In another European multi-centre study [19], problems with mood/apathy (as recorded on NMSS) were found among 88% of patients with PSP, while 36% had disturbances in perception/hallucinations. Patients with PSP were also significantly worse in terms of mood/apathy NMSS domain scores compared to patients with PD using comparative analyses (*p* = 0.0001) [19]. These findings were also mirrored in another comparative Italian multicentre study [2], where 67% of patients with PSP were found to have symptoms of apathy as reported on NMSS, while 73% had psychiatric problems. An apathetic neurobehavioral profile among patients with PSP has also been shown to predict a higher risk of disease-related mortality, further emphasising the need for better measurement tools to improve recognition and early intervention [63]. Another comparative study [15] demonstrated apathy and depression were more severe among patients with PSP compared to those who had PD, while apathy alone (without depression) was also more severe in PSP compared to MSA. Using QOLAS interview questionnaires, one study [12] found that among 27 patients with PSP, 54% reported symptoms of depression, 33% reported frustration, 29% anxiety, 21% sexual problems. A British longitudinal study [64] used several questionnaires, including the palliative care outcome scale (POS-S), to assess 15 patients with PSP and reported 13% of patients having hallucinations. An Italian study [16] which considered neuropsychiatric symptoms among outpatients with either PD (*n* = 155), PSP-Parkinson (PSP-P) (*n* = 11) and PSP-Richardson Syndrome (PSP-RS) (*n* = 14), used multivariate logistical regression analysis and found that early phonological verbal fluency deficits helped distinguish patients with PSP-RS, whilst apathy was more likely to support a diagnosis of PSP-P. A comparative study conducted in Germany [17] demonstrated that patients with PSP-RS (*n* = 14) showed more neuropsychological and neurobehavioral deficits than those with PSP-P (*n* = 9).

### MSA

Among patients with MSA, mood and apathy symptoms were more common than other neuropsychiatric manifestations. One study [2] found that among 34 patients with MSA, 65% had symptoms of apathy, while 79% had psychiatric problems as reported on the NMSS. This finding was mirrored in another prospective study which also used the NMSS among 172 patients with MSA (*n* = 76 MSA-P, *n* = 96 MSA-C) [65], and found that 88% reported disturbances in mood/apathy (88% MSA-PC, 89% MSA-C), 74% reported depressive symptoms (76% MSA-P, 72% MSA-C) and 45% reported symptoms of anxiety (40% MSA-P, 50% MSA-C) [65]. Furthermore, 70% of patients with MSA had reported a loss of interest in surroundings (76% MSA-P, 66% MSA-C), while 67% reported a lack of motivation (70% MSA-P, 65% MSA-C). Hallucinatory symptoms and delusional thought disorders were scarcely reported among patients with MSA in this study (3.5% [3.9% MSA-P, 3.1% MSA-C] and 0.6% [1.3% MSA-P, 0% MSA-C] respectively) [65]. Similarly, among 17 patients with MSA included in one study [64], only 29% reported hallucinatory symptoms on the POS-S.

### CBD

Among 11 patients with a clinical syndrome of CBD included in the Italian multicentre comparative study [2], 64% of patients were reported to have symptoms of apathy, while 55% had psychiatric problems using NMSS.

### DLB

The prevalence of neuropsychiatric and behavioural symptoms in patients with DLB varied among published studies. In one large multicentre study [2], 86% of the 14 patients with DLB had symptoms of apathy as reported on NMSS, while 93% reported psychiatric problems. In another retrospective study conducted in Japan [66] the prevalence of prodromal NMS was surveyed using standardised questionnaires among 34 patients with DLB (*n* = 31 ‘probable’, *n* = 3 ‘confirmed’) presenting to a memory clinic who were matched by age, gender and MMSE scores to 30 control patients. 24% of patients with DLB had depressive symptoms, while 26% had apathy, 26% had anxiety symptoms, and 12% had a mood disturbance [66]. When the onset of NMS preceding cognitive impairments were explored among patients with DLB, 32% were found to have preceding symptoms of depression (mean duration − 4.5 ± 10.7 years). Certain NMS were actually found to be more common after the onset of cognitive impairments among patients with DLB; including symptoms of anxiety in 38% (0.3 ± 2.9 years); moodiness in 29% (0.8 ± 2.5 years) and a lack of motivation in 62% (2.4 ± 4.1 years) [66].

## Sleep disturbances

Sleep problems are reported frequently by patients with atypical parkinsonian syndromes, including rapid eye movement (REM) sleep disorder (RBD) in alpha-synculeinopathies [67] and excessive daytime sleepiness (EDS).

### PSP

Sleep disturbances are particularly common among patients with PSP, where the prevalence reported in the literature ranges between 77–100% [2, 18, 19, 64]. In one study conducted in Western China [18], all patients with PSP demonstrated sleep/fatigue symptoms on NMSS, and the frequency and severity of sleep/fatigue symptom domains on NMSS were found to be significantly higher compared to that seen in healthy controls (*p* < 0.05). A similar finding was reported in another multi-centre European study [19], where most patients with PSP (92%) reported sleep/fatigue symptoms, with significantly worse sleep/fatigue NMSS domain scores compared to patients with PD using comparative analyses (*p* = 0.0007). Similarly, another European multicentre study [2] reported sleep disturbances among 77% of patients with PSP using NMSS, while another longitudinal study [64] identified that 73% of patients with PSP exhibited sleepiness, while 40% exhibited difficulty sleeping using standardised questionnaires and POS-S scores.

### MSA

Among patients with MSA, the prevalence of sleep symptoms varied greatly in the published literature. Using POS-S scores, one longitudinal study [64] reported a prevalence of 82% for both excessive sleepiness symptoms and symptoms of difficulty sleeping. Meanwhile, two other studies [2, 65] used the NMSS to evaluate sleep symptoms: one study reported sleep disturbance in 68% of patients with MSA [2] while the other reported excess daytime sleepiness in 53% of patients with MSA(57% MSA-P, 50% MSA-C) [65].

### CBD

One study [2] considered the prevalence of sleep symptoms among patients with a clinical syndrome of CBD, wherein the reported prevalence was 36% using NMSS.

### DLB

Sleep disturbances were relatively common among patients with DLB, with a wide repertoire of sleep-related symptoms being identified. In general, sleep disturbances among patients with DLB were reported to be 79% in one European multicentre study [2] using NMSS, while another Japanese study [66] found sleep rhythm disturbances to occur in 62% of patients with DLB using a standardised survey of NMS. The latter group [66] also explored the prevalence of RBD features and found that 35% of DLB patients had abnormal limb movements during sleep, while 27% experienced nightmares. RBD is now included as a core criterion for the diagnosis of DLB [61] because it occurs frequently among autopsy-confirmed cases compared with non-DLB dementia cases (76% vs 4%) [68]. When the onset of NMS preceding cognitive impairments was explored among DLB patients, antecedent features of an anREM-sleep disorder were found to be common in DLB: crying or shouting in sleep preceded cognitive impairments in 77% of patients (mean duration − 4.9 ± 9.5 years); preceding abnormal limb movements sleep in 47% (− 3.9 ± 9.1 years); preceding nightmares in 38% (− 2.5 ± 8.0 years), and preceding sleep rhythm disturbances in 82% (− 0.5 ± 5.5 years) [66]. Sleep duration of less than 5 h per day or the presence of hypersomnolence have also been significantly associated with DLB amongst patients with various dementia syndromes [62].

## Fatigue

Fatigue, as a symptom distinct from sleep problems, is widely reported among patients with atypical parkinsonian syndromes.

### PSP

Fatigue symptoms were particularly common among patients with PSP, wherein two studies [2, 64] both demonstrated a prevalence of 80% using either NMSS or POS-S scores respectively.

### MSA

Among patients with MSA, fatigue symptoms were also frequently reported, with one longitudinal study [64] observing a prevalence as high as 88% using POS-S scoring. Two studies used the NMSS and observed the prevalence of fatigue at 82% [2] and 61% (62% MSA-P, 60% MSA-C) [65]. Another study [69] utilised the Fatigue Severity Scale (FSS) and found nearly a quarter of MSA patients reported fatigue symptoms using this tool.

### CBD

One European multicentre study [2] reported fatigue symptoms in more than half of patients with a clinical syndrome of CBD as measured by the NMSS.

### DLB

The same multicentre study [2] also reported the prevalence of fatigue symptoms using NMSS in more than 70% of patients with DLB.

## Gastrointestinal symptoms

Among the autonomic symptoms experienced by patients with atypical parkinsonian syndromes and PD, gastrointestinal symptoms including constipation appeared to be the most common.

### PSP

Gastrointestinal symptoms, as reported on NMSS, were particularly common among patients with PSP, wherein three studies reported a prevalence between 80–86% [2, 18, 19]. One comparative study [18] found that the frequency and severity of gastrointestinal symptom domains on NMSS were significantly higher among patients with PSP compared to healthy controls (*p* < 0.05), while another [19] reported worse gastrointestinal NMSS domain scores among patients with PSP compared to those with PD using comparative analyses (*p* < 0.0001). Using the POS-S score and structured questionnaires, another study [64] also found that 60% of patients with PSP reported constipation symptoms, while only 7% reported nausea.

### MSA

The prevalence of gastrointestinal symptoms among patients with MSA varied depending on the study and the type of assessment tools used. Two studies used the NMSS: one [2] reported gastrointestinal symptoms to occur in 82% of patients with MSA; while the other [65] observed difficulty swallowing occurring in 53% of patients with MSA (57% MSA-P, 50% MSA-C) and constipation in 55% (66% MSA-P, 46% MSA-C). Using standardised questionnaires and POS-S scoring, another study [64] reported nausea to occur in 41% of patients with MSA and constipation in 77%. Similarly, one comparative study [13] involving both patients with MSA-P and MSA-C (*n* = 95 and *n* = 48 respectively) observed a prevalence of constipation of 73% in all patients with MSA.

### CBD

One comparative study [2] reported gastrointestinal symptoms to occur in 64% of patients with a clinical syndrome of CBD as reported on NMSS.

### DLB

Among patients with DLB, one comparative study [2] reported gastrointestinal symptoms to occur in 86% of patients using NMSS. Using standardised surveys of non-motor PD symptoms, another study [66] found that 47% of patients with DLB had constipation while 53% experienced antecedent features of constipation prior to memory loss when the onset of NMS preceding cognitive impairments was explored (mean duration − 9.4 ± 16.9 years).

## Urinary symptoms

Voiding difficulties and urge urinary incontinence are some of the most important features of autonomic dysfunction seen in MSA [70], but such symptoms of urinary dysfunction are also frequently reported among patients with other types of atypical parkinsonian syndromes.

### PSP

74% of patients with PSP in the study from western China [18] reported symptoms of urinary dysfunction on NMSS. Conversely, in another European multi-centre study [19], 92% of patients with PSP had symptoms of urinary dysfunction and had significantly worse urinary dysfunction NMSS domain scores compared to patients with PD using comparative analyses (*p* = 0.0001). Another study [2] also using the NMSS, found 53% of patients with PSP had urinary symptoms.

### MSA

There is, not surprisingly, more work on urinary dysfunction symptoms in patients with MSA. One study [71] explored post-void residual (PVR) volumes and found that this was significantly higher among patients with MSA compared to those with PD or PSP. PVR volumes were also demonstrated to correlate strongly with symptoms of urinary dysfunction (e.g. incomplete micturition, weak urine stream, and nocturia), other autonomic features, NMS and activity of daily living scores among patients with MSA [71]. Perhaps unsurprisingly, the study also demonstrated a negative relationship between PVR and renal function [71]. Among studies using questionnaires, the prevalence of urinary dysfunction symptoms varied between 71–91% [64, 65] in patients with MSA. Another study from China [13] reported nocturia in 80% of patients with MSA, urinary urgency in 78%, and urinary incontinence in 70% using standardised questionnaires. Using the NMSS, one study [2] reported a prevalence of urinary symptoms of 91%.

### CBD

There is very little research into urinary dysfunction among patients with a clinical syndrome of CBD. Only one study [2] considered urinary symptoms among patients with the condition and reported a prevalence of 55%.

### DLB

Using the NMSS, one study [2] reported urinary dysfunction symptoms to occur in 79% of patients with DLB. Meanwhile, another study [66] utilised a standardised survey of NMS and only observed urinary incontinence symptoms among 26% of patients with DLB. Perhaps unsurprisingly, these symptoms were subsequently found to be more common (53%) among patients with DLB after the onset of cognitive impairments (2.4 ± 4.8 years) [66], since the impairment of cognition or dementia are risk factors for the development of urinary incontinence.

## Cardiovascular symptoms

Cardiovascular autonomic symptoms such as neurogenic orthostatic hypotension and orthostatic dizziness are important contributors to recurrent falls. Such NMS are commonly associated with MSA, although less commonly may also be reported by patients with other atypical parkinsonian syndromes.

### PSP

Two studies considered the prevalence of cardiovascular symptoms among patients with PSP, wherein one study [2] reported a prevalence of 13% for postural hypotensive symptoms using the NMSS. The other study [72] utilised standardised questionnaires to compare autonomic symptoms among patients with either PSP (*n* = 32), MSA (*n* = 38), PD (*n* = 26) or age-matched healthy controls (*n* = 27), along with five autonomic function tests (deep breathing, Valsalva manoeuvre, tilt-table testing, sympathetic skin response, pupillography, and 24-h ambulatory blood pressure monitoring). Other than pupillography, no other laboratory-based autonomic function tests distinguished one patient group from another, alone or in combination [72].

### MSA

The prevalence of cardiovascular symptoms and orthostatic hypotension has varied greatly among published literature considering patients with MSA. Two studies [2, 65] utilised the NMSS and reported postural symptoms in 31–55% of patients with MSA, while one study [65] observed light-headedness symptoms to occur in 70% of patients (65% MSA-P, 74% MSA-C), while falls due to fainting occurred in 16% using NMSS (16% MSA-P, 16% MSA-C). Another study [33] considered both patients with MSA-P and MSA-C and found that 57% of patients had confirmatory evidence of postural hypotension, while 79% had orthostatic symptoms. Conversely, another study [13] only reported a prevalence of orthostatic hypotension among 30% of patients with MSA. One study [73] compared the prevalence of orthostatic hypotension among patients with either Parkinson’s disease (*n* = 1125) or atypical parkinsonian disorders (MSA *n* = 26, CBD *n* = 14, PSP *n* = 26), and found this to be most prevalent among patients with MSA (81%) compared to all other groups (PD 18%, DLB 31%, PSP 26%, CBD 7%).

### CBD

One study [2] considered the prevalence of orthostatic postural symptoms among patients with a clinical syndrome of CBD and found that none of the patients reported these symptoms.

### DLB

Using the NMSS, one study [2] demonstrated a prevalence of postural symptoms due to orthostatic hypotension of 21% among patients with DLB. This was similar to another study [66] which conducted a standardised survey of non-motor PD symptoms and reported a prevalence of orthostatic dizziness symptoms of 24% in patients with DLB. Orthostatic dizziness symptoms were also common after the onset of cognitive impairments, with 29% of patients exhibiting symptoms (mean duration 0.0 ± 2.1 years after the onset of cognitive impairment) [66]. The prevalence of cardiovascular symptoms among patients with PSP was 18% in a study from western China [18], and 50% in the European multi-centre study [19] using NMSS.

## Sexual dysfunction

Sexual dysfunction is the least commonly discussed autonomic manifestation among patients with atypical parkinsonian disorders, yet appears to be one of the most commonly reported patient symptoms, particularly in MSA and PSP.

### PSP

The prevalence of sexual dysfunction symptoms varied greatly among patients with PSP. Using the NMSS, one study [18] reported the prevalence of such symptoms to be as high as 93%, with the frequency and severity of sexual dysfunction symptom domains on the NMSS being scored significantly higher among those with PSP compared to PD (*p* < 0.05) or healthy controls (*p* < 0.05). Conversely, another European multi-centre study [19] found the prevalence of sexual dysfunction to only be present in 18% of patients with PSP using the NMSS, which was similar to a third study [12] which reported sexual dysfunction symptoms in 21% of patients with PSP using QOLAS.

### MSA

One study [65] considered the prevalence of sexual dysfunction among patients with MSA in China using the NMSS, and reported a prevalence of 73% (70% MSA-P, 75% MSA-C). In another European study, genital hyposensitivity was reported by 56% of the women with MSA and 9% controls (*p* < 0.0001) [74].

## Olfactory dysfunction

As is the case in PD, symptoms of anosmia may precede the development of core diagnostic features in DLB. More recent research suggests smell and taste disturbances may also be reported by patients who develop other atypical parkinsonian syndromes albeit not as commonly as that observed in PD.

### PSP

Olfactory testing has previously shown merit in differentiating patients with PSP or idiopathic PD [75]. In one study [76], it was found that the olfactory scores of people with autopsy-confirmed PSP (mean UPSIT score 21.9 ± 9.4) were higher compared to those with PD (mean 13.8 ± 5.3), but lower than controls (mean 26.8 ± 6.8). Furthermore, patients who had a mixed PSP-PD pathology (mean 16.9 ± 4.5) had scores that reflected this where their score values were in between those observed with pure PSP or pure PD pathology.

### MSA

Using the NMSS, one study [65] found anosmia or taste disturbances to occur in 23% of patients with MSA (MSA-P 28%, 20% MSA-C). Olfactory deficits are also thought to be less prominent in MSA than PD [77].

### CBD

It is thought that odour identification deficits in CBD may be attributed to perceptual deficits [78] in the context of generalised executive dysfunction, as opposed to a pure olfactory loss as observed in cases of PD (where the primary pathology originates from olfactory structures such as the olfactory bulb) [79].

### DLB

In one study [66], using standardised surveys of NMS it was found that 41% of patients with DLB had anosmia symptoms. When the onset of NMS preceding cognitive impairments was explored, antecedent features of anosmia preceded memory loss in 65% of patients on average 3 years before cognitive impairment [66].

## Pain

Symptoms of pain have a significant impact for many patients with atypical parkinsonian syndromes, yet is mostly underreported. Studies have reported such symptoms to affect between 40–80% of patients. Symptoms of pain may affect the limbs, neck or back [80], and can be either neuropathic or musculoskeletal in origin or, in some cases, related to dystonia [81].

### PSP

Symptoms of pain among patients with PSP were considered by two studies: one study [2] observed such symptoms to occur in 40% of patients using NMSS, while the other [64] observed a prevalence of 60% using POS-S.

### MSA

Pain symptoms are frequently reported in patients with MSA, where one study [2] reported a prevalence of 71% using the NMSS, while another [64] reported a prevalence of 88% using POS-S. However, a further study found that pain intensity as recorded on the visual analogue scale did not differ significantly in 136 patients with either PD or MSA [82].

### CBD

One study [2] assessed pain symptoms in patients with a clinical syndrome of CBD and reported a prevalence of 36% using NMSS. In a pooled meta-analysis [80] of three studies involving 55 patients, the prevalence of pain symptoms was found to be slightly lower at 25%.

### DLB

Half of the patients with DLB that were included in one study [2] reported pain symptoms using the NMSS. In a previous systematic review, the prevalence of such symptoms was found to range widely between 25–70% of cases [81].

## Other autonomic, respiratory and speech problems

Autonomic symptoms are a common feature of alpha-synucleinopathies, where they are a distinguishing feature for prodromal Lewy body disease [83] as well as a core criterion in the diagnosis of MSA [70]. Respiratory problems including nocturnal stridor, hypopnea and apnoea are common in MSA. [84]

### PSP

One study [2] used the NMSS and reported skin disturbances (including seborrhoea and hyperhidrosis) in 17% of patients with PSP and respiratory symptoms (such as dyspnoea, cough or stridor) in 21%.

### MSA

Skin disturbances (including seborrhoea and hyperhidrosis) were reported among 24% of patients with MSA using NMSS, while respiratory symptoms (such as dyspnoea, cough or stridor) were reported among 35% using NMSS [2]. Two additional studies explored the prevalence of excess saliva production among patients with MSA using NMSS: one study [65] reported a mean prevalence of 30% in patients with MSA-P and MSA-C (although found that this was more common among MSA-P patients [45% MSA-P, 17% MSA-C]), whilst the other study [13] observed a prevalence of 71% among patients with MSA. Stridor has been previously reported to occur among nearly two-thirds of patients with MSA [84]. Both obstructive sleep apnoea (OSA) and central sleep apnoea (CSA) may affect patients with MSA: in one French study involving 45 patients with MSA, 31% had OSA and 9% had CSA [85].

### CBD

Among patients with a clinical syndrome of CBD, one study [2] used the NMSS and found skin disturbances (including seborrhoea and hyperhidrosis) to occur in 27% and respiratory symptoms (such as dyspnoea, cough or stridor) in 9%.

### DLB

One study [2] found that the prevalence of skin disturbances (including seborrhoea and hyperhidrosis) and respiratory symptoms (such as dyspnoea, cough or stridor) among patients with DLB were both 29%. Hypersalivation symptoms were reported in 21% of DLB patients in one study [66], although this was more common (32% of patients) after the onset of cognitive impairments (0.5 ± 2.1 years). Meanwhile, the study also observed symptoms of increased sweating in 15% of patients, and when the onset of NMS preceding cognitive impairments was explored it was found that 29% had preceding increased sweating (mean duration − 6.5 ± 18.7 years) [66].

## Discussion

Some studies have demonstrated that certain non-motor characteristics might help support a diagnosis of a particular atypical parkinsonian syndrome or, in some cases, point away from a diagnosis of PD. In a large study from the United Kingdom, it was found that a third of patients who were initially diagnosed with PD subsequently had their diagnoses revised to PSP after two years [22]. Fluid intelligence and digit recall scores in PSP patients were poorer than controls at 2.5–5 years before diagnosis, perhaps suggesting the existence of a long pre-diagnostic phase in PSP, with subtle changes in the cognitive function being an early biomarker [22]. A progressive non-fluent aphasia presentation can have either PSP or CBD as the underlying pathology [86]. Among these cases, the presence of early severe dysarthria, relatively selective white matter atrophy at presentation, and a greater rate of change in the brainstem measured by longitudinal imaging may be useful in these cases for differentiating underlying PSP from CBD pathology during life [86]. Meanwhile, features of nocturnal inspiratory stridor, inspiratory sighs, severe dysphonia, cold hands and feet, Raynaud’s phenomenon, excessive snoring and emotional incontinence are all considered ‘red flags’ for MSA as outlined by the European MSA study group [87].

In recent years, Braak’s gut-origin hypothesis [88] has gained significant traction as driving PD pathology where it is thought that alpha-synuclein aggregates (Lewy pathology) first accumulate in the submucosal plexus of the gut and subsequently spread to the brain [89]. This is reflected by the prominence of gastrointestinal symptoms observed in PD; with constipation being one of the most recognised and earliest symptoms of the disorder. We found no statistically significant difference in the prevalence of constipation between PSP (56%) [64, 72], MSA (60%) [13, 64, 65, 72], DLB (47%) [66], CBD (63%) [2] and PD groups (50%) (*p* = 0.20) [3]. All groups had a significantly higher prevalence compared to healthy age-matched controls (20.4%) (*p* < 0.001) [90], hence constipation as an NMS may not reliably distinguish PD from other atypical parkinsonian syndromes in later stages, and warrants further research to evaluate its prevalence in prodromal phases of the atypical parkinsonian syndromes as studied in PD.

On the other hand, hyposmia or anosmia appears to be relatively specific to PD, occurring as a prodromal feature in approximately 75% of cases [3]. While olfaction scores may also decrease with advancing age [91], severe olfactory loss in a patient presenting with a neurodegenerative syndrome likely predicts underlying PD pathology rather than an atypical parkinsonian syndrome. Silviera-Moriyama et al [92] found that only 20% of PSP patients reported problems with smell, compared to a prevalence of up to 97% of PD patients reporting either hyposmia or anosmia [93]. Shill and colleagues [76] suggested a sensitivity of 93.4% and specificity of 64.7% for PSP among patients presenting with parkinsonism and normosmia. When compared to other atypical parkinsonian syndromes, Wenning and colleagues [94] also reported a marked olfactory loss among patients with PD, in contrast to milder impairments in MSA, and normal olfaction in cases of CBD.

Sleep problems are common in movement disorders and are often reported early in the disease course [95]. Both EDS and RBD are common in parkinsonian syndromes and result in significant morbidity [95]. We found EDS prevalence in PSP to be significantly higher (94.3%) [18, 19, 64] than PD (33.9%) [3] and normal controls (10.5%) (*p* ≤ 0.001) [3]. In the context of a patient presenting with dementia, concomitant early symptoms of EDS were more likely to suggest DLB, rather than AD or behavioural variant frontotemporal dementia (bvFTD), both of which had a lower prevalence of EDS [96].

The risk of developing a neurodegenerative disorder with accumulating alpha-synuclein pathology in the brain (synucleinopathy) is high among people who develop idiopathic RBD [97]. The estimated risk of developing a synucleinopathy is 30% at 3 years, rising to 66% at 7.5 years [97]. While both polysomnography-proven RBD and positive responses to a screening test for RBD are listed as biomarkers in the Movement Disorders Society (MDS) research criteria for prodromal PD [98], the prevalence of RBD is similar among PD (synucleinopathy) and PSP (tauopathy) [99], suggesting perhaps a more downstream cause of parkinsonism driving the development of RBD rather than its primary neuropathology. Sleep disturbances (defined as any of the following symptoms: EDS, insomnia, restless legs or RBD symptoms) were more prevalent in PSP (76.6%) [2] and MSA (67.6%) [2], compared to PD (35.4%) (*p* < 0.001) [3].

Autonomic dysfunction is highly prevalent in idiopathic RBD, with greater degrees of dysfunction at baseline being associated with phenoconversion from RBD to DLB [100]. Urinary symptoms and orthostatic hypotension, the two commonest symptoms of autonomic dysfunction, are also listed as part of the diagnostic criteria for MSA [70, 101]. We noted a greater prevalence of urinary dysfunction (not limited to urinary incontinence) was present in patients with MSA (79.7%) [2, 64, 65, 71, 72] or PD (79.9%) [102], compared to PSP (49.3%) [2, 18, 19, 32, 64, 71, 72], DLB (42%) [2, 66] or CBD (53.8%) (*p* < 0.001) [2].

Neurogenic orthostatic hypotension as a manifestation of autonomic failure is most frequently associated with MSA, but may also be observed in other atypical parkinsonian syndromes as well as in early PD [103]. Consistent with this, we found the prevalence of orthostatic hypotension to be significantly higher in MSA (49%) [2, 13, 33, 65, 72, 73] compared to PD (14.7%) (*p* < 0.001). The prevalence of orthostatic hypotension symptoms did not significantly differ between DLB (24%) [2, 66], PSP (15.6%) [72] or PD (14.7%) (*p* = 0.168) [2]. Orthostatic hypotension surprisingly was not reported in a case series of pathologically confirmed CBD [104].

Due to the latency of onset of orthostatic hypotension symptoms, the point prevalence of orthostatic hypotension in MSA and PD are usually based on overall disease duration [105]. In MSA, the median latency for developing significant orthostatic hypotension is typically 2 years, while in some cases patients with PD may only exhibit symptoms after 10 years [104]. Furthermore, while orthostatic hypotension is classically defined as a fall in systolic blood pressure of > 20 mmHg on standing, patients with MSA historically required at least a > 30 mmHg fall in systolic blood pressure to fulfil previous diagnostic criteria [101] (although more recently this has since been modified to meet the standardised definition of a > 20 mmHg fall in systolic blood pressure in the latest Movement Disorder Society MSA criteria [70]). Hence, among asymptomatic MSA patients, the true incidence of such symptoms may be underreported [105] if the previous criteria are used. While earlier autonomic dysfunction is associated with more aggressive disease progression and shorter survival in PD [106], its relationship with atypical parkinsonian syndromes is less clear and warrants further evaluation.

Neuropsychiatric disturbances including apathy, anxiety, depression and hallucinations are frequently described in both PD [107] and atypical parkinsonian disorders [108], of which depression is the most frequently reported disturbance [107, 108]. Apathy (defined as a lack of feeling, emotion, interest or concern) is considered by some to be a separate syndrome in patients with PD, as half of the patients with apathy do not suffer from concomitant depression or cognitive impairments [109]. We found the prevalence of apathy was significantly higher in MSA (48%) [2, 15, 65] and DLB (56%) [2, 66] compared to PD (35%) [110] (*p* < 0.001). Apathy prevalence was similar among CBD (43%) [2, 8] (*p* = 0.479) and PSP (37%) [12, 15, 16, 19] (*p* = 0.914) compared to PD [110]. We found anxiety symptoms were more prevalent in MSA (45.3%) [2, 65] compared to PD (20%) [107] (*p* < 0.001), while there were no significant differences between PSP (17.3%) [12, 16] (*p* = 0.659) or DLB (26.5%) [66] (*p* = 0.419) when compared to PD [107].

Depressive symptoms are an independent predictor of quality of life in both PD [111] and atypical parkinsonism [112]. Comorbidities such as anxiety, memory problems, hallucinations, sleep disturbances and postural hypotension are more common among depressed PD patients [113]. We found the prevalence of depression to be significantly higher among MSA (69.1%) [15, 65] and PSP (45%) [12, 15, 16, 19], compared to DLB (23.5%) [66], CBD (33.3%) [8], PD (36.6%) [3] or healthy controls (14.9%) (*p* < 0.001) [3].

Psychosis in late-stage PD is a common neuropsychiatric complication that imposes significant challenges for caregivers and is a risk factor for nursing home placement [114]; while also significantly contributing to morbidity, mortality and overall quality of life among patients [115]. Psychosis may also affect patients with PSP, MSA, CBD and DLB [2]. The disease burden associated with psychosis in DLB is significantly higher compared to other types of dementia or DLB without psychosis [116]. While delusions, hallucinations, and thought disorders are core clinical features of psychosis [117], transient or isolated hallucinatory symptoms (such as hallucinations of presence or passage) may not always signify psychosis. Approximately 10% of patients with PD retain insight into their hallucinations [107], which mostly only last a few seconds [118]. In DLB, one of the four core diagnostic features are recurrent visual hallucinations, which are usually well formed and detailed [61]. In keeping with this, we found the prevalence of hallucinations (visual or auditory) to be significantly higher in DLB (61.8%) [119], compared to PSP (16.6%) (*p* = 0.025) [64] and CBD (22.2%) [8], PD (27%) [107], MSA (5.8%) [64, 65] or healthy controls (1%) (*p* < 0.001) [102], while the prevalence of delusions was significantly higher in DLB (51.2%) [2] compared to MSA (< 1%) [2, 65], CBD (11.1%) [2, 8] or PD (15.8%) (*p* < 0.001) [107]. While hallucinations and delusions can be present due to iatrogenic causes (i.e. drug-induced), they may also occur spontaneously as part of an organic spectrum of neuropsychiatric symptoms in PD [120]. Apart from DLB, hallucinations and delusional disorders have not been reported to occur frequently in other atypical parkinsonian syndromes [121], but we found this to be reported in a significant proportion of PSP patients.

Treatment options for NMS among atypical parkinsonian usually follow the standard clinical practice as that would be managed in PD, for example, pharmacotherapeutic treatments in the management of neuropsychiatric symptoms (e.g. depressive or psychotic symptoms) or orthostatic hypotension (Table 5). More recently, there has been interest in the efficacy of pharmacotherapeutic interventions in managing NMS in atypical parkinsonian syndromes; for example, a randomised control trial is currently underway exploring the efficacy of rivastigmine (currently licensed for use in mild-to-moderate Alzheimer’s dementia) on cognitive impairments and apathy in PSP [122]. Furthermore, novel drugs such as Rho Kinase (ROCK) inhibitors (e.g. Fasudil) are currently under investigation in an open-label single-arm interventional trial among patients with tauopathies (including PSP and CBD). Protein levels of Rho-associated protein kinases (ROCK1 and ROCK2) are thought to be increased in PSP/CBD brains, with previous animal studies demonstrating how pharmacologic inhibition of Rho kinases in neurons diminished detergent-soluble and -insoluble tau through a combination of autophagy enhancement and tau mRNA reduction [123], potentially paving the way for a new means of treating such tauopathies.

**Table 5:** Treatment options for the NMS in atypical parkinsonian syndromes

Symptom	Treatment options
Constipation	1. Increase fibre in the diet and rehydration 2. Lactulose or Macrogol or Senna
Excessive day time sleepiness	1. Investigate for and treat any underlying cause first 2. Modafinil (avoid in pregnancy)
Orthostatic hypotension	1. Review and address pharmacological causes 2. Support stocking and increase salt/fluid intake 3. Fludrocortisone 4. Midodrine
Pain	1. Investigate for and treat any underlying cause first 2, Pregabalin or Gabapentin or Duloxetine
Urinary urgency or incontinence	1. Investigate for and treat any underlying cause first 2. Trospium or Solifenacin for overactive bladder 3. Intermittent self-catheterization if underactive bladder
Restless legs	1. Treat iron deficiency and look for secondary causes 2. Ropinirole or Pramipexole or Rotigotine
Anxiety	1. Cognitive behavioural therapy 2. Low dose anti-depressant (SSRI or TCA)
Depression	1. Cognitive behavioural therapy 2. Low dose anti-depressant (SSRI or TCA)
Hallucinations and delusions	1. Investigate for and treat any underlying cause first such as UTI causing delirium 2. Quetiapine if no cognitive impairment 3. Rivastigmine if coexistent dementia
Psychosis	1. Investigate for and treat any underlying cause first such as medication overdose 2. Quetiapine or Clozapine
Impulse control disorders	1. Withdrawal of any dopamine agonists (DA) (done gradually to avoid DA withdrawal syndrome)
REM sleep behavior disorder	1. Clonazepam 2. Melatonin
Dementia	1. Investigate for and treat any reversible cause first 2. Cholinesterase inhibitor (e.g. Rivastigmine)
Sialorrhoea	1. Using handkerchief to wipe excess saliva 2. Speech and language therapy 3. Glycopyrronium or Atropine drops 4. Botulinum toxin injections into salivary glands

Most research into NMS among patients with atypical Parkinsonian syndromes have generally relied on repurposing existing scales used in other conditions. For example, some studies extended the use of NMSS [19, 65], whilst others either used modified or improvised versions of questionnaires validated in PD [2, 66], or symptom scales designed for use in palliative care [64]. Some scales have been validated for use in atypical parkinsonian syndromes, for example, PSP rating scales which exist to assess both motor and some NMS features [124], although these are generally limited in the range of NMSS covered. Despite the similarity in the range of NMS observed among patients with PD and atypical parkinsonian disorders, there are clear differences in the relative prevalence of the different NMS [2]. Development of an analogous scale to encompass NMS among the atypical parkinsonian syndromes may therefore help to fully appraise comparisons between differing conditions and determine specific factors which may predict an individual condition. However, given the heterogenous nature of NMS and its wide repertoire, the development of such scales may potentially pose a technical challenge in terms of encompassing all possible symptoms which may manifest in the wide repertoire of atypical parkinsonian syndromes.

### Limitations of the study

Compared to studies with PD, most studies of NMS in atypical parkinsonian syndromes are limited by their relatively small sample sizes (typically < 50 cases), and the heterogeneous case mix of confirmed and probable cases (wherein many cases in the included studies were not pathologically confirmed). For instance, among those with corticobasal syndrome, only half of the cases were accompanied by confirmatory histological findings for CBD [125]; whilst PSP, AD and TDP-43 pathology [126] may mimic the clinical syndrome. Many of the studies we included utilised standardised questionnaires (e.g. NMSS) which are validated for use in PD, but not atypical parkinsonian syndromes, limiting their generalisability or the accuracy of observed clinical findings, warranting the need for dedicated larger-scale studies with disease-specific validated questionnaires to confirm our results.

## Conclusions

NMS are common among atypical parkinsonian disorders and may be underreported by patients unless systemically sought out in clinical practice. Evaluating NMS in atypical parkinsonian syndromes is essential to providing a holistic clinical care plan and appropriate pharmacological treatments for these patients to help with their unmet needs.

*SSRI* selective serotonin reuptake inhibitor, *TCA* tricyclic antidepressant, *DA* dopamine agonists

### Supplementary Information

Below is the link to the electronic supplementary material.

Supplementary file1 (DOCX 962 KB)
