# Correction to: Neurological update: non-motor symptoms in atypical parkinsonian syndromes

**DOI:** 10.1007/s00415-023-12139-6

**Published:** 2023-12-27

**Authors:** Piriyankan Ananthavarathan, B. Patel, S. Peeros, R. Obrocki, N. Malek

**Affiliations:** 1 Department of Neurology, National Hospital for Neurology and Neurosurgery, Queen Square, London, UK; 2 Department of Neuroinflammation, Institute of Neurology, University College London, 1st Floor, Russell Square House, 10-12 Russell Square, London, WC1B 5EH UK; 3 Department of Neurology, Queen’s Hospital, Romford, Essex UK

**Correction to: Journal of Neurology (2023) 270:4558–4578** 10.1007/s00415-023-11807-x

The original version of this article unfortunately contained a mistake. The corrected detail is provided below:

In section “CBD”, fourth paragraph, which previously read:

Visual dysfunction appears to be another common problem among patients with CBD [37, 42, 45, 48, 51, 55, 56], and is included in the diagnostic criteria for the disease [51, 57, 58]. Although several studies [37, 45, 48] did not find any gross visual impairment on formal testing, one review [41] discussed how this might be due to confounding factors such as limb apraxia, motor deficits or co-existent executive dysfunction which could limit the ability to formally test visuospatial function.

Should read:

Visuospatial dysfunction appears to be another common problem among patients with CBD [37, 42, 45, 48, 51, 55, 56], and is included in the diagnostic criteria for the disease [51, 57, 58]. Although several studies [37, 45, 48] did not find any gross visual impairment on formal testing, one review [41] discussed how this might be due to confounding factors such as limb apraxia, motor deficits or co-existent executive dysfunction which could limit the ability to formally test visuospatial function.

