# Supplementary material for: Neurological update: non-motor symptoms in atypical parkinsonian syndromes
Source: J Neurol. 2023 Jun 15;270(9):4558–78. doi: 10.1007/s00415-023-11807-x (PMC10421812; doi:10.1007/s00415-023-11807-x)
Supplement: Supplementary file 1 — Supplementary file1 (DOCX 962 KB) [file 415_2023_11807_MOESM1_ESM.docx]

**Supplementary Table 1**: Critical appraisal of papers – Risk of bias analysis of papers included in this systematic review (papers listed according to year of publication).

|  | **Risk of bias** | | | | |
| --- | --- | --- | --- | --- | --- |
| ***Study*** | ***Selection*** | ***Detection*** | ***Analysis*** | ***Reporting*** | ***Attrition*** |
| *Schrag, A et al (2003)* |  |  |  |  |  |
| *O’ Sullivan, S (2008)* |  |  |  |  |  |
| *Reiman, M (2010)* |  |  |  |  |  |
| *Colisomo, C et al (2010)* |  |  |  |  |  |
| *Srulijes, K et al (2011)* |  |  |  |  |  |
| *Ha, A et al (2011)* |  |  |  |  |  |
| *Higginson, I et al (2012)* |  |  |  |  |  |
| *Chiba, Y et al (2012)* |  |  |  |  |  |
| *Ikeda, C et al (2014)* |  |  |  |  |  |
| *Ou, R et al (2016)* |  |  |  |  |  |
| *Zhang, L et al (2017)* |  |  |  |  |  |
| *Radicati, F et al (2017)* |  |  |  |  |  |
| *Pelicano, C et al (2017)* |  |  |  |  |  |
| *Santangelo, g et al (2018)* |  |  |  |  |  |
| *Lee, Y et al (2018)* |  |  |  |  |  |
| *Du, J et al (2018)* |  |  |  |  |  |
| *Barcelos, L et al (2018)* |  |  |  |  |  |

Low risk Unclear risk High risk
